# The first chromosome-level genome assembly of *Entomobrya proxima* Folsom, 1924 (Collembola: Entomobryidae)

**DOI:** 10.1038/s41597-023-02456-w

**Published:** 2023-08-16

**Authors:** Jianfeng Jin, Yuxin Zhao, Guoqiang Zhang, Zhixiang Pan, Feng Zhang

**Affiliations:** 1 Department of Entomology, College of Plant Protection, Nanjing Agricultural University, Nanjing, 210095 China; 2 School of Life Sciences, Taizhou University, Taizhou, 318000 China

**Keywords:** Genome, Comparative genomics

## Abstract

The Entomobryoidea, the largest superfamily of Collembola, encompasses over 2,000 species in the world. However, the lack of high-quality genomes hinders our understanding of the evolution and ecology of this group. This study presents a chromosome-level genome of *Entomobrya proxima* by combining PacBio long reads, Illumina short reads, and Hi-C data. The genome has a size of 362.37 Mb, with a scaffold N50 size of 57.67 Mb, and 97.12% (351.95 Mb) of the assembly is located on six chromosomes. The BUSCO analysis of our assembly indicates a completeness of 96.1% (n = 1,013), including 946 (93.4%) single-copy BUSCOs and 27 (2.7%) duplicated BUSCOs. We identified that the genome contains 22.16% (80.06 Mb) repeat elements and 20,988 predicted protein-coding genes. Gene family evolution analysis of *E. proxima* identified 177 gene families that underwent significant expansions, which were primarily associated with detoxification and metabolism. Moreover, our inter-genomic synteny analysis showed strong chromosomal synteny between *E. proxima* and *Sinella curviseta*. Our study provides valuable genomic information for comprehending the evolution and ecology of Collembola.

## Background & Summary

Collembola is one of the most abundant and diverse groups of terrestrial arthropods, containing more than 9,400 known species in the world, with at least 410 million years of evolutionary history^1^. Their distributions range from the Arctic to Antarctica, and from a 1,760 m deep cave to a 5,000 m high mountain^2^. Collembola exhibit a wide range of feeding habits, including consuming litter, fungi, pollen, algae, leaves, and roots^3–5^. Furthermore, numerous collembolan species demonstrate sensitivity to environmental factors, making them suitable indicators for assessing soil ecotoxicology and monitoring environmental changes^6^.

Entomobryoidea is the largest superfamily of Collembola, containing approximately 2,000 species found in various terrestrial ecosystems, and is an essential member of soil communities, mainly occurring in humid forests and grasslands^1^. This monophyletic group is a key player in collembolan phylogeny and is divided into two families: Orchesellidae and Entomobryidae^7^, with *Entomobrya* (Rondani, 1861) being the type genus of Entomobryidae and some species of this group are important pests of mushrooms^8,9^. To obtain a high-quality genome of Collembola, a combination of second and third-generation sequencing is necessary. However, due to their small body size (<10 mm), a single collembolan individual does not provide sufficient DNA/RNA for library construction, requiring the pooling of multiple individuals for sequencing. Furthermore, several generations of feeding are necessary to reduce heterozygosity, further increasing the challenge of obtaining a high-quality genome. To date, 51 collembolan genomes have been reported (as of July 2023 from NCBI), with only seven species sequenced using third-generation sequencing and four genomes at the chromosome level. However, the majority of these genomes assembled from short reads have poor assembly quality, with scaffold N50 lengths of less than 100 kb.

To enhance our knowledge of the evolution and ecology of Entomobryoidea, we obtained a chromosome-level genome of *Entomobrya proxima* (Folsom, 1924) through the combination of PacBio long reads, Illumina short reads, and Hi-C data. We annotated repeats, non-coding RNAs (ncRNAs), and protein-coding genes (PCGs), and conducted gene family evolution analysis. Moreover, we explored the interspecific chromosomal variation between the two Entomobryidae species, *E. proxima* and *Sinella curviseta*. The high-quality genome of *E. proxima* is an important milestone in our understanding of Entomobryoidea and will contribute to the study of collembolan evolution and ecology.

## Methods

### Sample collection and sequencing

The *E. proxima* samples used in this study were collected in June 2020 from the Purple Mountain in Nanjing, China (32.06 °N, 118.83 °E). Adult individuals were collected by an entomological aspirator, washed in phosphate-buffered saline, and instantly frozen using liquid nitrogen. A total of 100, 30, 50, and 50 individuals were used for PacBio, genome survey, Hi-C, and transcriptome sequencing, respectively. To obtain the requisite amount of DNA/RNA for sequencing, we utilized a pooled extraction approach, in which multiple individuals were combined prior to nucleic acid extraction.

Genomic DNA and RNA from pooled specimens were extracted using the DNeasy Blood & Tissue Kit and TRIzoTM Reagent, following the manufacturer’s instructions. PCR-free short-read libraries of 150 bp paired-end read with a 350 bp insert size were generated using the Truseq DNA PCR-free Kit. The Hi-C sequencing was carried out by digesting extracted DNA with the Mbol restriction enzyme. We utilized the Illumina NovaSeq 6000 platform to sequence all short-read libraries. A library with a 30 kb-insert size was prepared using the SMRTbell Template Prep Kit 1.0-SPv3 for PacBio sequencing. To generate the library, we used the PacBio Sequel II platform, which employs the PacBio CLR mode. Berry Genomics (Beijing, China) carried out all library construction and sequencing. Finally, we obtained 292.18 Gb of sequencing data, comprising 206.73 Gb (570.50×) of PacBio reads, 45.89 Gb (126.62×) of Illumina reads, 29.83 Gb (82.32×) of Hi-C data, and 9.73 Gb of transcriptome data (Table 1). The raw PacBio long reads had a scaffold N50 and an average length of 29.41 and 24.08 kb, respectively.

**Table 1:** Statistics of the sequencing data used for genome assembly.

Libraries	Insert sizes (bp)	Clean data (Gb)	Sequencing coverage (x)
Illumina	350	45.89	126.62
PacBio	30 Kb	206.73	570.50
Hi-C	350	29.83	82.32
RNA	350	6.91	—
Iso-Seq	5 Kb	2.82	—

### Genome assembly

We performed quality control on raw Illumina data using BBTools v38.82^10^, which included the “clumpify.sh” script to remove duplicate reads. We trimmed low-quality reads using the “bbduk.sh” script, which consisted of base quality trimming of both ends (>Q20), length filtering (>15 bp), polymer A/G/C trimming (>10 bp), and correction of overlapping paired reads.

**Fig. 1:**
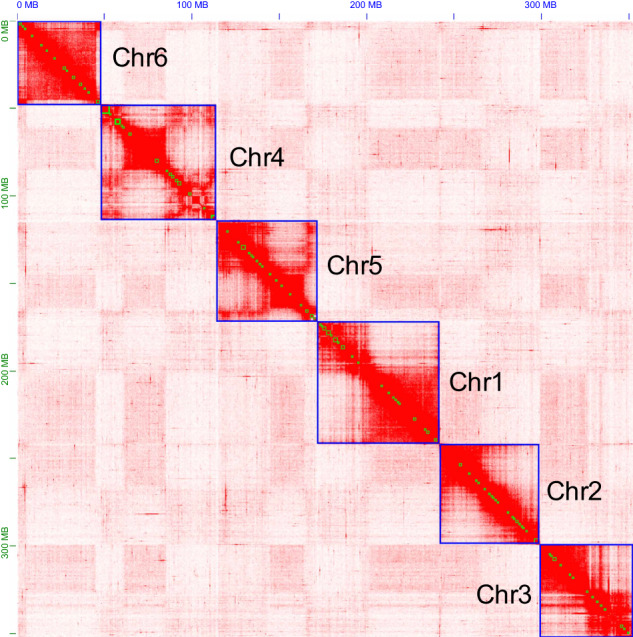
Genome-wide chromosomal heatmap of *Entomobrya proxima*, with each chromosome and contig framed in blue and green, respectively.

The primary assembly of PacBio long reads was generated using Flye v2.8.3^11^, with a minimum overlap of 3,000 between reads. We then performed one round of self-polishing on the assembly. NextPolish v1.1.0^12^ was used to polish the primary assembly with Illumina reads in two rounds. Redundant contigs were eliminated using Purge_Dups v1.2.5^13^ with a haploid cutoff set at 50 for identifying contigs as haplotigs (“-s 50”). We used Minimap2 v2.17^14^ for read mapping during the above “remove haplotigs” and “short-read polishing” steps. We aligned Hi-C reads to the assembly after performing quality control using Juicer v1.6.2^15^. Subsequently, we anchored the contigs onto chromosomes using 3D-DNA v180922^16^. To ensure accuracy, we manually reviewed and corrected any errors using Juicebox v1.11.08^16^. We detected possible contaminants using MMseqs. 2 v11^17^, which performed BLASTN-like searches based on the NCBI nucleotide and UniVec databases with a sequence identity of 0.8 (‘–min-seq-id 0.8’). Additionally, we utilized blastn (BLAST + v2.11.0^18^) against the UniVec database to specifically detect vector contaminants. We considered that sequences with over 90% hits in the aforementioned database likely contained contaminants. Sequences with over 80% hits were checked again via online BLASTN analysis in the NCBI nucleotide database. We then removed potential bacterial and human contamination from the assembled scaffolds. Most of the identified contaminants were bacterial, including Rickettsia and Sphingobacterium. The final chromosome-level genome assembly of *E. proxima* had a size of 362.37 Mb, comprising 461 scaffolds and 2,007 contigs, with the scaffold and contig N50 sizes of 57.67 Mb and 0.44 Mb, respectively. Among them, 1,542 contigs (97.12%, 351.95 Mb) were anchored into six chromosomes with lengths ranging from 48.13 to 69.80 Mb and the GC content was 35.93% (Table 2; Fig. 1). The genome size and GC content of *E. proxima* (362.37 Mb and 35.93%) were smaller than that of *S. curviseta* (363.44 Mb and 37.52%) (Table 3).

**Table 2:** Genome assembly statistics for *Entomobrya proxima*.

Assembly	Total length (Mb)	Number scaffolds/contigs (chromosomes)	Scaffold/contig N50 length (Mb)	GC (%)	BUSCO (n = 1,013) (%)			
					C	D	F	M
Flye	687.89	6,402/6,510	0.40/0.38	35.71	97.1	16.3	0.8	2.1
NextPolish	687.38	6,260/6,260	0.38/0.38	35.71	97.4	16.6	0.6	2.0
Purge_Dups	363.21	16,531,683	0.53/0.52	35.93	96.1	3.6	0.9	3.0
3D-DNA	363.09	694/2,240 (6)	57.70/0.44	35.93	96.1	2.7	0.7	3.2
Final	362.37	461/2,007 (6)	12.53/6.54	35.93	96.1	2.7	0.7	3.2
Transcript	87.82	27,393/27,406	4.38/4.38 Kb	39.77	95.3	28.5	0.9	3.8

C: complete BUSCOs; D: complete and duplicated BUSCOs; F: fragmented BUSCOs; M: missing BUSCOs.

**Table 3:** Genome assembly and annotation statistics for two chromosome-level assemblies of *Entomobrya proxima* and *Sinella curviseta*.

Characteristics	*E. proxima*	*S. curviseta*
Genome assembly		
Size (Mb)	362.37	363.44
Number of scaffolds	461	476
Number of chromosomes	6	6
Scaffold N50 length (Mb)	57.67	62.78
GC (%)	35.93	37.52
BUSCO completeness (%)	96.1	96.8
Protein-coding genes		
Number	20,988	22,485
Mean gene length (bp)	4,610.4	4,517.3
BUSCO completeness (%)	96.9	98.3
Repetitive elements		
Size (Mb)	80.06 (22.16%)	51.44 (14.16%)
DNA transposons (Mb)	6.60 (1.77%)	4.97 (1.34%)
SINEs (kb)	10.23 (0.00%)	35.67 (0.01%)
LINEs (Mb)	5.87 (1.77%)	3.13 (0.87%)
LTRs (Mb)	18.82 (5.20%)	14.77 (4.08%)
Unclassified (Mb)	43.03 (11.88%)	22.97 (6.32%)
Number of ncRNA	391	583
rRNA	23	55
miRNA	23	34
snRNA	42	58

### Genome annotation

We employed RepeatModeler v2.0.3^19^ and the “-LTRStruct” LTR discovery pipeline to generate a repeat library. We then merged this library with the Repbase-20181026^20^ and Dfam 3.1^21^ into a custom library. RepeatMasker v4.1.2-p1^22^ was utilized to identify repetitive elements in the *E. proxima* genome by aligning it against the custom library. RepeatMasker analysis revealed that the *E. proxima* genome contains approximately 22.16% (80.06 Mb) repetitive elements, comprising unclassified elements (11.88%), LTR elements (5.20%), DNA transposons (1.77%), LINE (1.77%), simple repeats (0.61%), as well as other elements (Table S1). Notably, the percentage of repetitive elements in *E. proxima* (22.16%) was higher than that in *S. curviseta* (14.16%) (Table 3).

We used Infernal v1.1.4^23^ and tRNAscan-SE v2.0.9^24^ to identify ncRNAs and tRNAs, respectively. The tRNAscan-SE with the script “EukHighConfidenceFilter” was used to filter low-confidence tRNAs. We identified 391 ncRNAs in the genome of *E. proxima*, which contained 23 ribosomal RNAs, 23 microRNAs, 42 small nuclear RNAs, 152 transfer RNAs, seven ribozymes, and 144 other ncRNAs (Table S2). The number of ncRNAs in *E. proxima* (391) was lower than that in *S. curviseta* (583) (Table 3).

We annotated the PCGs using MAKER v3.01.03^25^ based on three strategies, containing *ab initio* predictions, transcribed RNA, and homologous proteins. To maximize *ab initio* predictions, we employed the BRAKER v2.1.6^26^ and GeMoMa v1.7.1^27^ tools, using transcriptome and protein evidence, and combined their results as the *ab initio* input for MAKER. HISAT2 v2.2.0^28^ was employed for producing transcriptome alignments. BRAKER used Augustus v3.3.4^29^ and GeneMark-ES/ET/EP 4.68_lic^30^ as predictors and automatically trained them from RNA-seq alignments and reference proteins mined from OrthoDB10 v1 database^31^. GeMoMa employed protein homology and intron position conservation to predict genes, using the parameters “GeMoMa.c = 0.4 GeMoMa.p = 10” and protein sequences from seven species (*Cloeon dipterum* (GCA_902829235.1), *Daphnia magna* (GCF_003990815.1), *Drosophila melanogaster* (GCF_000001215.4), a sexual strain of *Folsomia candida* (FCSH, GCA_020923455.1), *Rhopalosiphum maidis* (GCF_003676215.2), *Tribolium castaneum* (GCF_000002335.3), and *Zootermopsis nevadensis* (GCF_000696155.1)) (Table 4). We used RNA-seq alignments produced from HISAT2 to perform genome-guided assembly by StringTie v2.1.6^32^. Additionally, protein sequences from the same seven species employed in GeMoMa were incorporated as evidence of protein homology in MAKER. Finally, we predicted 20,988 PCGs in the *E. proxima* genome, with an average length of 4,610.4 bp. The average number of exons, introns, and CDS of each gene were 5.9, 4.9, and 5.7, respectively, and their corresponding mean length was 393.1, 481.1, and 281.8 bp, respectively (Table S3). BUSCO completeness of the protein sequences was 96.9% (n = 1,013), including 93.0% (942) single-copy, 3.9% (40) duplicated, 0.7% (7) fragmented, and 2.4% (24) missing BUSCOs, suggesting high-quality predictions.

**Table 4:** Species taxonomic information and accession code of all samples used in this study.

Species	Class	Order	Source
*Bombyx mori*	Insecta	Lepidoptera	NCBI (GCF_014905235.1)
*Catajapyx aquilonaris*	Diplura	Dicellurata	https://i5k.nal.usda.gov/
*Cloeon dipterum*	Insecta	Ephemeroptera	NCBI (GCA_902829235.1)
*Daphnia magna*	Branchiopoda	Cladocera	NCBI (GCF_003990815.1)
*Drosophila melanogaster*	Insecta	Diptera	NCBI (GCF_000001215.4)
*Entomobrya proxima*	Collembola	Entomobryomorpha	This study
FCDK	Collembola	Entomobryomorpha	NCBI (GCA_020920555.1)
FCSH	Collembola	Entomobryomorpha	NCBI (GCA_020923455.1)
*Holacanthella duospinosa*	Collembola	Poduromorpha	NCBI (GCA_002738285.1)
*Orchesella cincta*	Collembola	Entomobryomorpha	NCBI (GCA_001718145.1)
*Rhopalosiphum maidis*	Insecta	Hemiptera	NCBI (GCF_003676215.2)
*Sinella curviseta*	Collembola	Entomobryomorpha	10.6084/m9.figshare.7286231.v2
*Tomocerus qinae*	Collembola	Entomobryomorpha	10.6084/m9.figshare.17019872.v1
*Tribolium castaneum*	Insecta	Coleoptera	NCBI (GCF_000002335.3)
*Zootermopsis nevadensis*	Insecta	Isoptera	NCBI (GCF_000696155.1)

FCDK: a parthenogenetic strain of *Folsomia candida*; FCSH: a sexual strain of *Folsomia candida*.

We conducted the gene functional annotation search against the UniProtKB database using Diamond v2.0.11.149^33^ in sensitive mode with the parameters “–more-sensitive -e 1e-5”. We further employed eggNOGmapper v2.0.1^34^ and InterProScan 5.53–87.0^35^ to assign Gene Ontology (GO) and (KEGG, Reactome) pathway annotations and to identify protein domains. The InterProScan analyses included five databases: Pfam^36^, SMART^37^, Superfamily^38^, Gene3D^39^, and CDD^40^. The results predicted by the above tools were integrated to obtain the final gene function prediction. Genes with 13,129 GO terms, 13,129 KEGG pathways, 14,554 Reactome pathways, 4,393 Enzyme Codes, and 15,211 COG categories were assigned by integrating the InterProScan and eggNOG annotation results (Table S3).

### Phylogeny and gene family evolution

We inferred the orthology of PCG sequences across eight arthropod species, comprising one Diplura species (*Catajapyx aquilonaris*) and seven collembolan species (*E. proxima*, *S. curviseta*, *Orchesella cincta*, a parthenogenetic strain of *Folsomia candida* (FCDK), FCSH, *Tomocerus qinae*, and *Holacanthella duospinosa*) (Table 4). We used OrthoFinder v2.5.2^41^ to infer orthogroups (gene families), after eliminating redundant isoforms. For sequence alignment, we used the Diamond with ultra-sensitive mode (“-S diamond_ultra_sens”). We assigned 143,727 (89.6%) genes to 21,690 orthogroups, of which 3,924 were shared by all eight species and 2,252 were single-copy genes (Table S4). In *E. proxima*, 19,453 genes (92.7%) were contained in 12,705 gene families, of which 389 families and 1,886 genes were specific to this species.

**Fig. 2:**
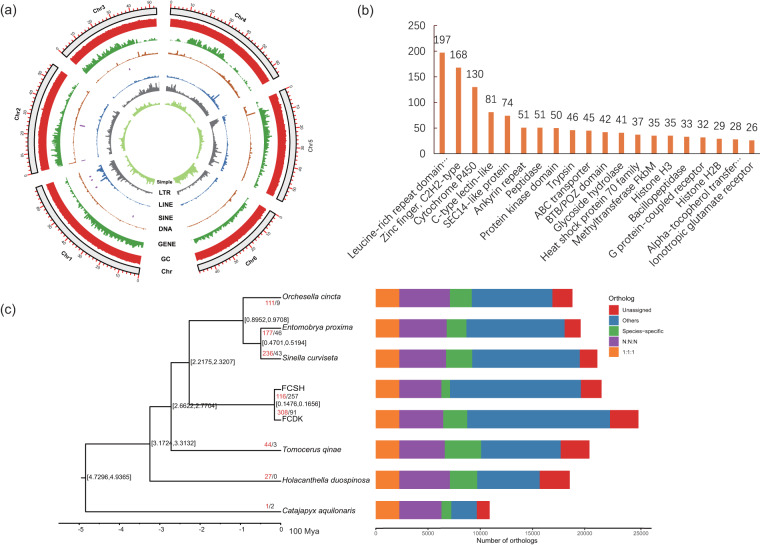
Genome characteristics, phylogeny, and gene family evolution of *Entomobrya proxima*. (**a**) From the outer ring to the inner ring are the distributions of chromosome length, GC content, gene density, TEs (DNA, SINE, LINE, and LTR), and simple repeats. (**b**) The top 20 gene families that significantly expanded in *E. proxima*. (**c**) The phylogeny and gene family changes among eight arthropod species, with node values representing the 95% highest probability densities of divergence times (unit, 100 Ma). The values labeled at terminals denote the number of significantly expanded and contracted gene families. “1:1:1” represents universal single-copy genes in all species, “N:N:N” represents multi-copy genes, “others” represents unclassified orthologues, and “unassigned” represents orthologues that cannot be assigned to any orthogroups.

We aligned single-copy orthologues using MAFFT v7.450^42^ with the high-accuracy LINS-I strategy. We then removed alignment gaps using trimAl v1.4.1^43^ with the “automated1” parameter. Finally, we reconstructed the phylogenetic tree on the single-copy orthologs using IQ-TREE v2.07^44^ with the LG site-homogeneous model. We employed MCMCTree in PAML v4.9j^45^ to predict species divergence time and nodes were calibrated using two fossils obtained from the PBDB database (https://www.paleobiodb.org/navigator/): the most common ancestor of Diplura and Collembola (407.6–410.8 Ma) and Entomobryomorpha (272.3–279.3 Ma). After filtering out 244 single-copy genes, we performed phylogenetic reconstruction using IQ-TREE based on 2,008 remaining genes. The analysis showed that *E. proxima* is closely related to *S. curviseta*, and the divergence time of these species was approximately 47.01‒51.94 Mya (Fig. 2c). Our phylogenetic results were consistent with previous studies, supporting Orchesellidae (*O. cincta*) as a sister group to Entomobryidae (*E. proxima*, and *S. curviseta*)^7,46,47^ (Fig. 2c).

**Fig. 3:**
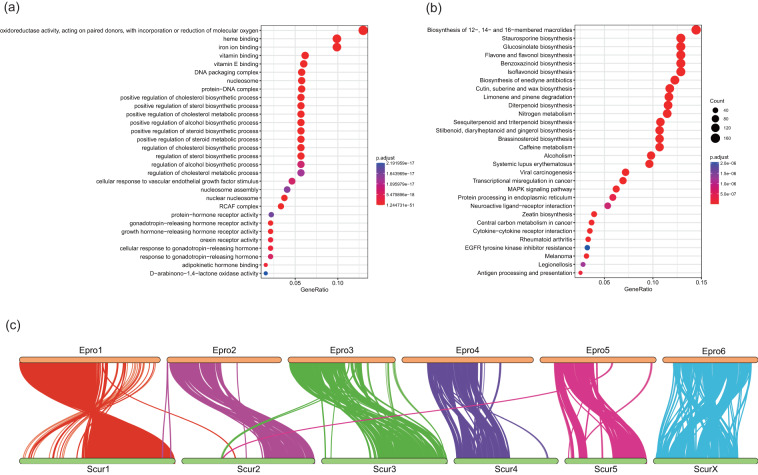
Gene enrichment, and chromosomal synteny. (**a**,**b**). GO and KEGG function enrichment of significantly expanded gene families. Only the top 30 categories are shown. (**c**). Chromosomal synteny between *Entomobrya* *proxima* (Epro) and *Sinella curviseta* (Scur).

We used CAFÉ v4.2.1^48^ to estimate the gene family evolution (expansion or contraction) based on the generated phylogenetic tree. We identified 1,351 expanded and 1,547 contracted gene families in *E. proxima*, including 223 gene families that underwent rapid evolution (177 expansions and 46 contractions). The significantly expanded families included the Leucine-rich repeat domain superfamily, zinc finger, Cytochrome P450, and other families that play important roles in the adaptive evolution of *E. proxima* (Table S5; Fig. 2b). Subsequently, we performed functional enrichment (GO and KEGG) analysis on PCGs from significantly expanded families using ClusterProfiler v3.10.1^49^ with default parameters. The enrichment of GO and KEGG in rapidly expanding families further indicates their function in the regulation of hormone levels, oxidoreductase activity, metabolic, and catabolic processes, among others (Fig. 3a,b). We uploaded the complete enrichment CSV tables to Figshare (10.6084/m9.figshare.23861901).

### Chromosome synteny

To investigate interspecific chromosomal evolution between *E. proxima* and *S. curviseta*, we used MMseqs. 2 with an e-value of 1e-5. Syntenic blocks were identified using MCScanX^50^ and the repeat content, gene density, and GC content on individual pseudochromosomes using TBtools^51^. Comparing the genomes of *E. proxima* and *S. curviseta*, we identified 341 syntenic blocks that contained 14,473 collinear genes (45.04%). The average number of genes per block was 42, while a notable 12.02% (41 blocks) contained over 100 collinear genes. We have uploaded the syntenic blocks results containing each block’s details to Figshare (10.6084/m9.figshare.23861901). Notably, our analysis revealed that the ChrX of *S. curviseta* shares strong chromosomal syntenic relationships with Chr6 of *E. proxima* (Fig. 3c). Other chromosomes (1–5) of *E. proxima* matched with their corresponding chromosomes in *S. curviseta*, although we did observe some regions lacking homology. These differences may be attributed to the significant divergence time (47.01–51.94 Mya) between the two species.

## Data Records

The raw sequencing data and genome assembly of *Entomobrya proxima* have been deposited at the National Center for Biotechnology Information (NCBI). The PacBio, Illumina, Hi-C, and transcriptome data can be found under identification numbers SRR15910088-SRR15910092^52–56^. The assembled genome has been deposited in the NCBI assembly with the accession number GCA_029691765.1^57^. Additionally, the results of annotation for repeated sequences, gene structure, and functional prediction have been deposited in the Figshare database^58^.

## Technical Validation

Two methods were used to evaluate the quality of the genome assembly. Firstly, we assessed assembly completeness using BUSCO v3.0.2^59^ with the reference arthropod gene set (n = 1,013). The final genome assembly showed a BUSCO completeness of 96.1%, consisting of 946 (93.4%) single-copy BUSCOs, 27 (2.7%) duplicated BUSCOs, 7 (0.7%) fragmented BUSCOs, and 33 (3.2%) missing BUSCOs. Secondly, we calculated the mapping rate as a measure of assembly accuracy. The mapping rates for PacBio, Illumina, and RNA reads were 99.93%, 88.83%, and 86.72%, respectively. These evaluations collectively reflect the high quality of the genome assembly produced in this study.

### Supplementary information

Supplementary table1

Supplementary table2

Supplementary table3

Supplementary table4

Supplementary table5

## Data Availability

No specific script was used in this work. All commands and pipelines used in data processing were executed according to the manual and protocols of the corresponding bioinformatic software.
